# Research progress of natural compounds in anti-liver fibrosis by affecting autophagy of hepatic stellate cells

**DOI:** 10.1007/s11033-021-06171-w

**Published:** 2021-02-20

**Authors:** Yongxiang Shu, Xuyou Liu, Haifeng Huang, Qi Wen, Jianchang Shu

**Affiliations:** Department of Gastroenterology, GuangZhou Red Cross Hospital, Jinan University, Guangzhou, 510220 China

**Keywords:** Liver fibrosis, Hepatic stellate cell, Natural compound autophagy, Apoptosis, Senescence

## Abstract

Chronic liver diseases caused by various pathogenesis are marked by inflammatory infiltration and wound healing reaction, while their normal regeneration ability is impaired. The unbalance between the generation and the degradation of extracellular matrix (ECM) leads to collagen accumulation and develops into liver fibrosis. Inflammation, oxidative stress, and autophagy interact closely in the pathogenesis of hepatic fibrosis. Reactive Oxygen Species (ROS) can not only stimulate Kupffer cells to release massive inflammatory factors, but induce autophagy. However, the latter may suppress inflammatory reaction by inhibiting proinflammatory complex formation directly, and removing damaged organelles or pathogenic microorganism indirectly. At present, effective anti-fibrosis drugs are still lacking. Previous studies have found various natural compounds enabled liver protection through anti-inflammatory, antioxidant, and other mechanisms. In recent years, autophagy, a vital life activity, has been found to be involved in the mechanism of liver fibrosis. As a new target, developing anti-liver fibrosis drugs that regulate the activity of autophagy is very promising. In this review, we summarize the latest studies about natural compounds in the treatment of liver fibrosis by regulating autophagy.

## Introduction

Chronic liver diseases caused by various causes is characterized by inflammatory infiltration and wound healing response, but the normal regeneration of liver cells is impaired. Finally, the imbalance between the generation and degradation of extracellular matrix leads to the accumulation of collagen and liver fibrosis (LF) [1]. Inflammation, oxidative stress and autophagy are closely related in the pathogenesis of LF. Unfortunately, due to the shortage of effective clinical treatment strategies [2, 3], exploring new therapeutic targets and drugs to reverse liver fibrosis remains an inevitable and tough task.

Autophagy, the hot research spot in recent years, has been shown to be involved in the pathogenesis of a lot of human diseases, such as cardiovascular diseases, neurodegenerative diseases, and tumors. Microtubule-associated proteins 1 light chain 3B (LC3B), p62/sequestosome1 (SQSTM1) and beclin-1 are the three widely used autophagy markers. LC3B will be successively processed and modified into two forms: LC3-I and LC3-II when autophagy is initiated. Cytoplasmic LC3(LC3-I) will enzymatically dissolve a small segment of polypeptide and change to the autophagosome membrane type (LC3-II). The autophagy level is, thus, estimated by the ratio of LC3-II/LC3-I [4]. Beclin-1 is one of the most important autophagy regulating genes. It forms a complex with Class III PI3K, mediating the expression of other autophagy proteins. P62, also known as SQSTM1, is a ubiquitin-like binding protein. P62, as a regulatory factor, is conjured to LC3 and participates in the formation of autophagosome. There is a negative correlation between the overall expression level of p62 in cells and autophagy activity [5].

Currently, an increasing number of studies have noticed the influence of autophagy in the pathogenesis of liver fibrosis, and the research field of HSCs autophagy is drawing more interest.

It has been a long history that natural products were used by humans as medicine to treat diseases. It also has been an important part of modern medicine to extract active ingredients from natural products. A large number of natural compounds have advantageous properties such as safety and effectiveness, some of which are used as a main source of anti-fibrotic drugs.

## The pathophysiological mechanism and therapeutic progress of hepatic fibrosis

The pathophysiological mechanism of liver fibrosis is very complex, involving a variety of cells and cytokines. Activation of HSCs is the central event of hepatic fibrosis, which has become the consensus [6]. HSCs activation is a pathological process characterized by a phenotypic transdifferentiation of HSCs to myofibroblastic-like cell. These “new” myofibroblasts exhibit the following characteristics: proliferation, chemotaxis, contractility, releasing inflammatory cytokines, loss of intracellular vitamin A droplets [7]. They synthesize various extracellular matrix components such as different types of collagens as well as proteins, all of which are implicated in the beginning of the fibrotic process. The mechanism of HSCs activation includes epithelial cell damage, changes in extracellular matrix, immune regulation, molecular imbalance mediated by membrane receptors and nuclear receptors, disorder of epigenetics transcription, cellular homeostasis and stress disorder which includes autophagy, and so on. These mentioned above are described in detail in a review published by Higashi T et al. [8]. In general, once HSCs become activated, they show “permanent” characteristics. However, it has been demonstrated that HSCs can return to a dormant state when the damage factors are removed, which may also explain the fact that liver fibrosis and cirrhosis can be reversed in some patients.

Although there have been a lot of studies on the pharmacotherapy of liver fibrosis, few of them have been used clinically, because the efficacy of these drugs is uncertain. At present, according to the mechanisms, treatment directions mainly include the following: (1) primary diseases therapy; (2) Using liver-protecting agent to reduce the damage; (3) Blocking myofibroblast activation, contraction and fibrosis formation; (4) Promoting apoptosis or reversal of activated stellate cells; (5) Stimulating matrix degradation [9].

## The role of autophagy in HSCs and LF

Autophagy is a catabolism process in which eukaryotic cells maintain cellular stability and organelle integrity by degrading intracytoplasmic components under stress. A certain degree of autophagy is essential to maintain the physiological function and normal metabolism. However, excessive enhancement or attenuation can lead to cell metabolism imbalance, thus causing the occurrence of diseases [10]. Previous studies have confirmed autophagy as a “double-edged sword” in a lot of diseases. Similarly, several recent studies revealed that the pathological mechanisms of many liver diseases may be autophagy dependent, such as hepatitis, hepatic steatosis, fibrosis, cirrhosis, and hepatocellular carcinoma [11].

At the moment, most studies tend to agree that when HSCs are stimulated, the autophagy level increases and provides energy for HSCs activation by promoting lipid degradation [12]. Therefore, enhanced autophagy is an accelerant for HSCs activation and hepatic fibrosis and reducing it to inhibit HSCs activation may be a therapeutic target. Indeed, previous studies by our group have shown that curcumin can inhibit autophagy and activity of LX-2 (a kind of immortalized HSC cell line) in vitro, and reduce autophagy level of liver tissue in vivo as well as the degree of liver fibrosis induced by CCl4 in rats, and it is speculated that the effect of curcumin on liver fibrosis is related to the inhibition of aHSCs autophagy [13, 14]. This is consistent with the view above.

However, some studies reported that the role of autophagy on LF and HSCs was diametrically opposed. For example, Seo HY et al. [15] concluded that after treatment of HSCs with phospholipase D1(PLD1), the production of I-type collagen (collagen I) had been decreased due to promoting autophagy. When autophagy was inhibited, the effect of PLD1 on collagen I had lost. What’s more, Liu, a member of our team, recently reported that overexpressing of microRNA-148a increased autophagic activity, which inhibited proliferation and promoted apoptosis in HSCs [16]. These illustrate that promoting autophagy may be a potential anti-fibrotic approach.

Although the certain reasons for these opposite conclusions are still unclear, some scholars believe that the different effects of autophagy on the fate of HSCs depend on the degree of autophagy, and the extent of autophagy depends on the intensity and duration of cellular stress. It is well known that either over-upregulation or down-regulation of autophagy is detrimental to cell survival. When quiescent HSCs are stimulated, autophagy promotes HSCs activation by altering lipid metabolism, and at this time, inhibition of autophagy may lead to decreased activity or apoptosis of aHSCs by interrupting energy supply. While, in activated HSCs (aHSCs), excessive autophagy can lead to HSCs senescence, autophagic cell death or apoptosis, collagen degradation and inflammation inhibition, alleviating liver fibrosis [17].

## Natural compounds exert anti-liver fibrosis effect via autophagy

Natural compounds can exert anti-fibrotic effects via a variety of mechanisms that mediate the activation of HSCs. Since autophagy is a hot topic in life science, more and more researchers now focus on the role of autophagy in LF, trying to find new methods to treat it by intervening autophagy. Studies show that natural compounds can reduce LF through influencing the activity of HSCs by regulating inflammation, promoting senescence and autophagic cell death /apoptosis of HSCs. This article summarizes the newest studies to provide innovative ideas for scientific and clinical research (Table 1), and gives the molecular formulas and chemical structures of some of the natural compounds (Table 2).

**Table 1:** Effects of natural compounds on HSC autophagy and related mechanisms

Natural compounds	Direction of autophagy		Molecular mechanism	References
Trolline	Autophagy down-regulation		LC3-II/LC3-I ratio, Beclin-1, P62, Atg5, Atg7(−) → autophagy (−)	[18]
Shikonin			TGF-β1/Smads(−) → Beclin-1(−) →autophagy(−)	[20]
Taurine			PPARα(−) → autophagy(−)	[22]
Dihydroartemisinin (DHA)	Autophagy up-regulation	Inhibiting inflammation	ROS-JNK 1/2(+) → autophagy(+) → inflammation reaction(−)	[24]
Catalpol			LC3-II、Beclin-1(+) P62、IL-6、IL-8、TNF-ɑ(−) →autophagy(+)	[26]
Methyl Helicterate (MH)		Inducing HSCs apoptosis	JNK(+) PI3K/Akt/mTOR(−) →autophagy(+)	[29]
Purple pitanga extract			autophagy/mitochondrial autophagy (+) → apoptosis (+)	[30]
Caffeine			IER1-ɑ(+) → autophagy(+) → HSCs apoptosis(+)	[31]
Dihydroartemisinin (DHA)		Inducing HSCs senescence	autophagy (+) → GATA6 accumulation (+) → JNK1/2(+) → p53 and p16(+) → aHSCs aging (+)	[36]
Oroxylin A		Inhibiting HSCs viability	Atg3、Atg5、Beclin1/Atg6、Atg12、Atg14(+) → autophagy(+)	[37]
Caffeic acid phenethyl ester			AKT/mTOR(−) → autophagy(+)	[38]
Betulinic acid			MAPK/ERK(+) → autophagy(+)	[39]
Dihydrotanshinone I (DH I)			Hippo(−) → YAP/TEAD2(−) → autophagy(+)	[40]

Description: “+” means up-regulation or activation; “-” indicates decrease or inhibition; ”→” means regulation/mediation

**Table 2:** Molecular formulas and chemical structures of some natural compounds

Natural compounds	Molecular formula	Molecular weight	Chemical structure
Trolline	C12H13NO3	219.24	
Shikonin	C16H16O5	288.31	
Taurine	C2H7NO3S	125.15	
Dihydroartemisinin (DHA)	C15H24O5	284.35	
Catalpol	C15H22O10	362.33	
Methyl Helicterate (MH)	No data		
Purple pitanga extract	No data		
Caffeine	C8H10N4O2	194.19	
Dihydrotanshinone I (DH I)	C18H14O3	278.30	
Betulinic acid	C30H48O3	456.70	
Oroxylin A	C16H12O5	284.26	
Caffeic acid phenethyl ester(CAPE)	C17H16O4	284.31	

### Natural compounds inhibiting HSCs activation by down-regulating autophagy

Trolline is a kind of isoquinoline alkaloid isolated from the flowers of *Trollius chinensis* Bunge (Ranunculaceae). Bai [18] et al. found that trolline significantly reduced CCl_4_-induced SD rats’ liver injury and collagen deposition. Additionally, They found that trolline inhibited HSC-T6 autophagy by down-regulating the ratio of LC3-II/LC3-I, Beclin-1 and p62 which were the major proteins of autophagy. What’s more, Atg5 and Atg7, the two key genes involved in conversion LC3-I to LC3-II, also decreased in the level of transcription. Therefore, they speculated trolline may partially inhibit the activation of HSCs by downregulating autophagy. However, their studies lack a control group that includes autophagy agonist as well as related signaling pathways.

Shikonin is a kind of natural product extracted from the roots of *Lithospermum erythrorhizon* and has anti-inflammatory, anti-tumor and antioxidant characteristics [19]. Liu [20] et al. found that shikonin visibly alleviated CCl4 and BDL-induced LF in mice. In addition, shikonin obviously inhibited the activity of aHSCs and downregulated the level of autophagy. Since the TGF-β1/Smads signaling pathway initiates transcription of the Beclin-1 gene to regulate autophagy, they further checked TGF-β1 and phosphorylated Smad2/3 in both models and found both of them rising. Shikonin could downregulate their level in a dose-dependent manner. Therefore, they hypothesized that the mechanism of autophagy inhibition induced by shikonin may be partly due to the TGF-β1/Smads signaling pathway suppression.

Taurine is a sulfur-containing amino acid existing in a variety of human and animal tissue, which can reduce liver damage caused by several toxic substances such as alcohol, heavy metals, and chemicals [21]. Wang [22] et al. found As2O3 could stimulate the activation of LX-2 cells (a kind of immortalized and activated human HSC cell line) and the autophagy flux of them. Interestingly, the LX-2 cells activation was autophagy dependent, and taurine, though, could significantly relieve these effects. Previous studies indicated that the PPARα could regulate autophagy. Their further study demonstrated the autophagy flux was decreased by inhibiting PPARα. Therefore, they speculated taurine may have a therapeutic effect on LF by inhibiting HSCs autophagy, which was regulated by PPARα.

### Natural compounds that enhance autophagy in HSCs to alleviate LF

#### Natural compounds that inhibit inflammation by boosting autophagy in HSCs

Inflammation is involved in the whole process of LF. Inhibiting the release of inflammatory cytokines and improving the inflammatory microenvironment can even make the aHSCs convert to an inactivated state. Autophagy plays an important role in regulating the immune response and controlling inflammation. Specifically, autophagy controls inflammation through both directly inhibiting inflammatory complexes formation and indirectly suppressing it by removing damaged organelles or pathogenic microorganisms from cells. Thus, decreasing inflammatory response via regulating autophagy may have positive effects on LF.

It was reported that dihydroartemisinin (DHA), an effective antimalarial drug, could improve liver histopathology injuries and relieve LF in rat model [23]. Zhang [24] et al. found that DHA improved LF by restraining inflammatory responses. Meanwhile, autophagy level was upregulated in aHSCs. After down-regulating the autophagy of aHSCs with Atg5 siRNA, they found that DHA-induced anti-inflammatory effect was removed, while transfection of Atg5 plasmids could enhance the anti-inflammatory effect. Therefore, autophagy was considered indispensable for the anti-inflammatory effect of DHA in LF. Their further study revealed that H_2_O_2_, not O_2-,_ triggered autophagy activation of aHSCs. The anti-inflammatory effect of DHA is associated with autophagy activation via enhancing ROS-JNK1/2 signal pathway.

Catalpol, an iridoid glycoside extracted from traditional Chinese medicine *Rehmannia glutinosa*, has remarkable pharmacological effects such as antioxidant, anti-inflammatory, anti-diabetic, anti-tumor [25]. Liu [26] et al. found that catalpol could inhibit the secretion of fiber (α-SMA, fibronectin and α-1 procollagen) and inflammatory factors (IL-6, IL-8, TNF-α) and up-regulate the level of autophagy in aHSCs. However, inhibiting autophagy dramatically reduced the effect of catalpol on the inhibition of inflammatory factors release and collagen secretion from aHSCs. As a result, the function of catalpol in reducing LF through inflammation inhibition is autophagy dependent. Unfortunately, this study did not clarify the specific molecular mechanism between autophagy and inflammation regulation.

#### Natural compounds that induce HSCs apoptosis by increasing autophagy

Inducing aHSCs apoptosis is a strategy for the therapy of LF. A lot of natural compounds are cytotoxic to aHSCs. Contrary to well-known mechanisms of cell protection, autophagy also leads to cell death in specific cases, which is called autophagic cell death (ACD). ACD is the result of excessive cell autophagy, also being called II type programmed cell death. The main feature of ACD is that a large number of autophagy-lysosomes appear in cytoplasm, and most of the cytoplasmic material is degraded, but the nucleus remains integrity. Unlike apoptosis, ACD is generally independent of the activity of the caspase protein family [27]. Autophagy and apoptosis are two important cellular processes with complex and cross-protein networks. Bcl-2 has been identified as a central regulator of them. When nutrition is sufficient, Beclin-1 and Bax/BAK bind to Bcl-2 or Bcl-xL respectively, to prevent the initiation of autophagy and apoptosis. When under stress conditions, several mechanisms mediate the interruption of this interaction, thereby allowing them to be induced. In general, autophagy inhibits apoptosis by phagocytosis of pro-apoptotic caspases (e.g., caspase 8) and destruction of reactive mitochondria to prevent the release of cytochrome c. However, the autophagosome membrane serves as a platform for caspase-8 activation and promotion of apoptosis mediated by the intracellular death-inducing signaling complex. Thus, Over-intense autophagy can also trigger the occurrence of apoptosis [28]. Some natural compounds can induce apoptosis of aHSCs by inhibiting autophagy in the early stages of their activation, while others promote aHSCs ACD or apoptosis which depends on intense and sustained autophagy.

*Helicteres angustifolia* is a widely used Chinese herbal medicine for immune diseases and liver diseases treatment. Zhang [29] et al. identified methyl helicterate (MH) as the active component of this herbal medicine. They found that MH could induce apoptosis of HSC-T6 cells and significantly up-regulate autophagy level. After inhibiting autophagy, MH lost this ability. In contrast, enhancing autophagy promoted this effect. To clarify whether MH-induced autophagy is associated with apoptosis, they examined two important signaling pathways, JNK and mTOR, involved in hepatic fibrosis formation and HSCs activation. They found that MH remarkably induced the activation of JNK signaling pathway, while, the PI3K/Akt/mTOR pathway was inhibited, which resulted in autophagy activation and apoptosis of HSCs. Therefore, enhancing autophagy to induce aHSCs apoptosis is the essence of MH’s anti-hepatic fibrosis effect.

*Eugenia uniflora*, whose common name is pitanga or Brazilian cherry, is a plant widely distributed in South America. Its fruit has been proved to work as an antioxidant by inhibiting lipid peroxidation and scavenging free radicals. Denardin [30] et al. found that purple pitanga extract can reduce the viability of GRX cells (a kind of aHSCs line). They also found it could significantly induce autophagy and mitochondrial autophagy in GRX cells. However, enhanced autophagy not only impaired mitochondrial function, but declined mitochondrial content in the GXR cells, ultimately promoting GXR cells apoptosis via mitochondria-dependent pathways, which suggested that autophagy/mitochondrial autophagy induced by purple pitanga extract did not play a cytoprotective role, in contrast, leading to cell death. However, the molecular mechanism of the link between autophagy and apoptosis induced by purple pitanga extract remains unclear.

The protective effect of coffee and caffeine on the liver is drawing public attention. A number of studies have reported that regular intake of a certain amount of coffee can slow down the progression of chronic liver diseases. Li [31] et al. chose LX-2 cells as the research object and found that caffeine inhibited the vitality of LX-2 cells and induced apoptosis. They observed that caffeine induced endoplasmic reticulum (ER) stress and autophagy. Apoptosis of LX-2 cells was effectively weakened when autophagy was inhibited. After knocking down the IRE1-α gene, which regulates ER stress, the autophagic flux of LX-2 cells decreased. Based on the results, they believed that caffeine induces aHSCs apoptosis through autophagy, mediated by ER stress. However, this effect needs to be further verified in more HSCs lines, and the specific mechanism of autophagy leading to apoptosis is also worth further study.

#### Natural compounds that induce HSCs senescence by increasing autophagy

Previous studies also indicated a close relationship between autophagy and cellular senescence [32, 33], and aging HSCs have been found in liver fibrosis tissue [34]. Krizhanovsky [35] et al. reported that aging aHSCs secreted less ECM and promoted the reversal of LF. Thus, inducing aHSCs senescence may be a strategy to relieve LF.

Zhang [36] et al. found DHA-induced aHSCs senescence and attenuated LF in rats, and further confirmed the transcription factor GATA6 was the upstream signaling molecule for this effect. Indeed, their study showed that the accumulation of GATA6 could lead to the number of aging aHSCs rising. Besides, DHA could also induce autophagy and there was a close tie between the level of autophagy and GATA6. When autophagy was inhibited, the amount of GATA6 declining which led to the loss of senescent HSCs. Therefore, the HSCs aging induced by DHA is autophagy-dependent.

#### Natural compounds that inhibit HSCs viability by increasing autophagy

As mentioned above, most studies have shown that inhibition of autophagy can reduce the activity of aHSCs, and a lot of drugs exert anti-fibrosis effects through this mechanism. However, some drugs are found to inhibit HSCs activity by promoting autophagy and these drugs do not significantly increase the number of aging or apoptosis aHSCs, but inhibit their activity. Some of them work by enhancing autophagy to interfere with signaling pathways closely related to HSCs activation. Disappointedly, the mechanism of some other drugs remains unclear.

Oroxylin A is a natural mono-flavonoid in *Scutellaria*, a kind of Chinese herb, which has been proved to have anti-inflammatory, anti-angiogenic, antioxidative, and anti-tumor pharmacological activities. Chen [37] et al. found that Oroxylin A could decrease the deposition of ECM in CCl4-induced LF mice and down-regulated the expression of COL-1, α-SMA, PDGF-βR and TGF-βR which were all the molecular markers of aHSCs. Also, Oroxylin A upregulated the autophagy level in aHSCs remarkably. The anti-fibrotic effect of Oroxylin A completely lost after using autophagy inhibitor 3-MA, suggesting that this anti-fibrotic effect of Oroxylin A was triggered by activating autophagy. However, this study did not further explore the molecular mechanism of autophagy to reduce the activity of HSCs.

Caffeic acid phenethyl ester (CAPE) is one of the main medicinal components of *propolis*. CAPE is reported to have a variety of biological activities, such as anti-inflammatory, antioxidant, anti-tumor. Yang [38] et al. found that CAPE effectively attenuated CCl4-induced LF and inhibited the activity of HSCs. They observed the autophagy was upregulated in CAPE-treated HSC-T6 cells. To verify the role of autophagy, they used rapamycin (a kind of autophagy agonist) and 3-MA to treat HSC-T6 cells respectively with CAPE. The results showed that the rapamycin group further promoted autophagy and inhibited HSC-T6 cells activity, whereas 3-MA went the opposite direction. Meanwhile, they also confirmed that CAPE may attenuate liver fibrosis by inhibiting AKT/mTOR signaling pathway.

Betulinic acid (BA) is a natural triterpenoid, which is reported to have liver protective effect. Liu [39] et al. found that BA reduced the serum level of platelet-derived growth factor (PDGF) and hydroxyproline (HYP) in LF mice and the expression of α-SMA and collagen I. They also found that BA enhanced autophagy in both vivo and vitro. To verify whether this effect of BA was associated with autophagy, their further study showed that the effect mentioned above by BA was significantly relieved after using autophagy inhibitors, conveying that autophagy played a key role. Surprisingly, they validated that the role of BA in promoting autophagy was realized through inhibiting MAPK/ERK signaling pathway.

Dihydrotanshinone I (DHI), a lipophilic component extracted from *Salvia miltiorrhiza Bunge (Tanshen)*, has liver protection, anti-inflammatory, and weight-loss effects reported in previous studies. Ge [40] et al. found that DHI strongly repressed hepatic fibrogenic genes expression and in BDL-induced LF rats and LX-2 cells. In their study, DHI was also found to have a strong inhibitory effect on YAP/TEAD2 complex formation, the downstream molecular event of Hippo pathway which was closely associated with HSC activation. Simultaneously, they discovered that the autophagy flux was much higher in DHI treated HSCs. Based on these facts, they speculated that there might be some connection between autophagy and Hippo pathway. After YAP was knocked out, autophagy level in DHI treatment group was further increased and the anti-fibrotic effect was enhanced. Nevertheless**,** overexpression of YAP yielded the opposite results. In summary, DHI could alleviate LF by preventing the YAP/TEAD2 complex formation and stimulate autophagy. However, the underlying mechanism of YAP and autophagy requires further exploration.

## Conclusions and future prospective

Natural compounds have different effects on HSCs by regulating autophagy. Current studies indicate that the role of autophagy in LF is complicated. The effects of autophagy on HSCs may depend on its insensity and duration. A degree of autophagy enhancement helps activate quiet HSCs and maintain their activated phenotypes later on. While, in aHSCs, autophagy overexpression may lead aHSCs to be senescent, autophagic cell death or apoptosis [41]. As mentioned above, either over-strong or over-weak autophagy is a threat to the survival of cells. Therefore, enhancing or weakening autophagy both may play a role in promoting the inactivation or death of HSCs. The pharmacological effects of natural compounds are complex and may interfere with various life activities in cells. Therefore, to identify whether autophagy plays a major role and the underlying molecular mechanisms seems indispensable. Besides, the effects of changed autophagy level on other liver cells should not be ignored, because these non-stellate cells are also involved in the formation of LF, and the effect of autophagy on them is even opposite to that of HSC. For example, chronic liver cell damage is another key step in the development of LF, and autophagy has a protective effect on liver cells [42]. So, drugs targeting autophagy should be the focus of future research. Finally, a lot of anti-liver fibrosis drugs have poor clinical curative effect, possibly because basic research only focuses on the cellular or molecular level, which does help clarify pharmacological mechanisms but omits the complexity of biological systems [43]. Hence, it is of great significance to explore the metabolic differences between humans, animals, and cells.

Conclusively, natural compounds have a lot of advantages such as abundant reserves, wide sources, and relative safety. Developing natural drugs with anti-fibrosis effects has broad prospects and great significance.
